# Early Detection of Sepsis-Associated Encephalopathy Through Polymorphic Delta Waves in Single-Channel EEG: A Case Report

**DOI:** 10.7759/cureus.84628

**Published:** 2025-05-22

**Authors:** Juul Aben, Sjaak Pouwels, Annemarie Oldenbeuving

**Affiliations:** 1 Critical Care, Elisabeth-Tweesteden Hospital, Tilburg, NLD; 2 Intensive Care, Elisabeth-Tweesteden Hospital, Tilburg, NLD; 3 Surgery, Marien Hospital Herne, University Hospital of Ruhr University Bochum, Herne, DEU

**Keywords:** complications, delirium, electroencephalography, intensive care, monitoring, sepsis-associated encephalopathy

## Abstract

Acute encephalopathy is a rapidly developing brain disorder that is often seen in critically ill patients in the intensive care unit (ICU). This diagnosis can have multiple causes, of which sepsis is one of them. Sepsis-associated encephalopathy (SAE) indicates compromised brain function due to the accumulation of metabolic by-products or the cause of the sepsis itself. One distinctive electroencephalography (EEG) feature is the presence of polymorphic delta (PMD) waves, which can be present even before the patient has the clinical manifestations of sepsis. These PMD waves can be detected using a single-channel EEG device. In this case report, we present a patient who exhibited no overt clinical signs of sepsis or delirium, yet presented PMD waves and EEG slowing. This patient experienced two episodes of sepsis during hospitalization. This article serves as a case report to illustrate that sepsis may be detectable 'early' on a single-channel EEG due to the presence of distinct features like PMD waves.

## Introduction

The pathophysiology of sepsis-associated delirium and psychiatric changes has a multi-pathophysiological basis. Sepsis-associated encephalopathy (SAE) describes an acute cognitive dysfunction secondary to an infection that exists outside the central nervous system (CNS) [1,2]. Symptoms can vary from mild confusion to coma [1]. SAE is a common complication of sepsis, accounting for up to 70% of biochemical abnormalities, and stands as a major cause of delirium, even when the neurological examination of the septic patient appears normal [1-9].

Electroencephalography (EEG) has previously been shown in other research to show specific markers related to sepsis in critically ill patients [10-12]. One of them is the so-called polymorphic delta (PMD) waves [10-12]. These PMD waves can be measured by a single-channel EEG (DeltaScan, Prolira BV, Utrecht, The Netherlands) and are associated with delirium [4,10-12]. Previous research has also demonstrated specific EEG markers related to sepsis, such as an increase in delta waves and slowing and a decrease in alpha and theta activity in critically ill patients [13]. Our previous study [2] assessed the feasibility of using a single-channel EEG (DeltaScan) to detect delirium and also tried to investigate the concordance with other methods to assess delirium in critically ill patients in the ICU. One of the patients who was excluded from this study showed PMD waves and became septic a few days later. In this particular patient, PMD waves were observed days prior to the manifestation of clinical signs of sepsis. The question arises of whether single-channel EEG can be used to detect sepsis at a very early stage. The knowledge about SAE and the observations through the single-channel EEG suggest that sepsis may be identified early via this method, presenting a clinically feasible option to multiple-channel EEG [13]. Therefore, we report the clinical course of this patient as a case report.

## Case presentation

A 68-year-old man was hospitalized 17 days before the start of our initial study [2] due to gastric carcinoma for which he underwent subtotal gastric resection with Roux-en-Y reconstruction. Six days after surgery, he was admitted to the intensive care unit (ICU) due to incipient sepsis following a bowel perforation, probably due to latent diverticulitis. At the onset of sepsis, acute kidney injury (AKI) was also diagnosed.

The patient has been intubated and on a ventilator for 11 days. He had one pleural drain placed in his left lung due to pleural fluid after lobectomy, as well as two abdominal drains: one on the left and one on the right, after a laparoscopy for suturing a colon defect. The drains were in the epigastrium, near the transverse colon (closed defect), in the Douglasi cavum, and on the right paracolic. Medications were not given by nasogastric tube but were administered via a central venous catheter (CVC).

**Figure 1:**
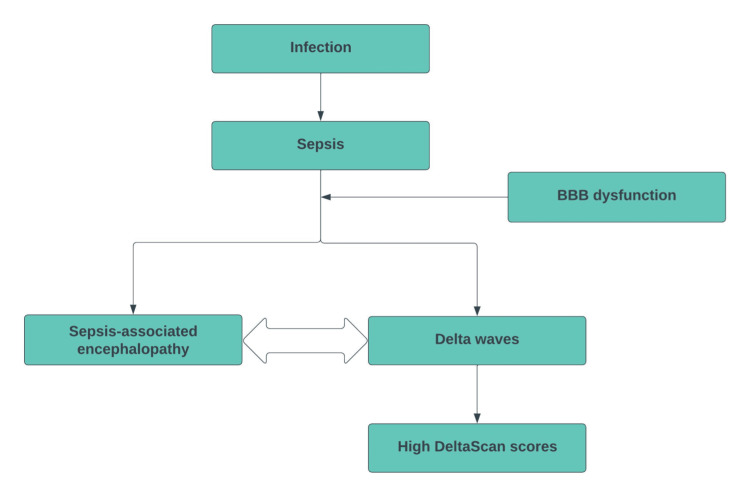
How sepsis can lead to high DeltaScan scores Note: This is a theoretical flowchart based on knowledge gathered from previous literature on sepsis-associated encephalopathy and delirium. We have written it ourselves, and it has not been published by anybody else. BBB: Blood-Brain Barrier

As part of our initial study [2], we started with the single-channel EEG measurements on the 11th day of his ICU stay. At that time, the clinical diagnoses of sepsis and AKI were no longer present according to the diagnosis, and the clinical values showed improvement. He satisfied the predefined inclusion criteria in the previous study [2]: (1) aged 50 years and older; (2) at least 24 hours of ICU admission; (3) intubated on the first day of the study measurements; and (4) have a sedation level using the Richmond Agitation Sedation Scale (RASS) between -3 and +4. The RASS score indicates the level of sedation required to ultimately obtain a delirium score, both from the CAM-ICU (Confusion Assessment Method for the ICU) and a DeltaScan EEG measurement. The data in this specific case showed contradictions: high DeltaScan scores but no (clinical) delirium, which may indicate that delirium was not measured on a single-channel EEG but possibly (early) sepsis through SAE (Figure 1).

**Figure 2:**
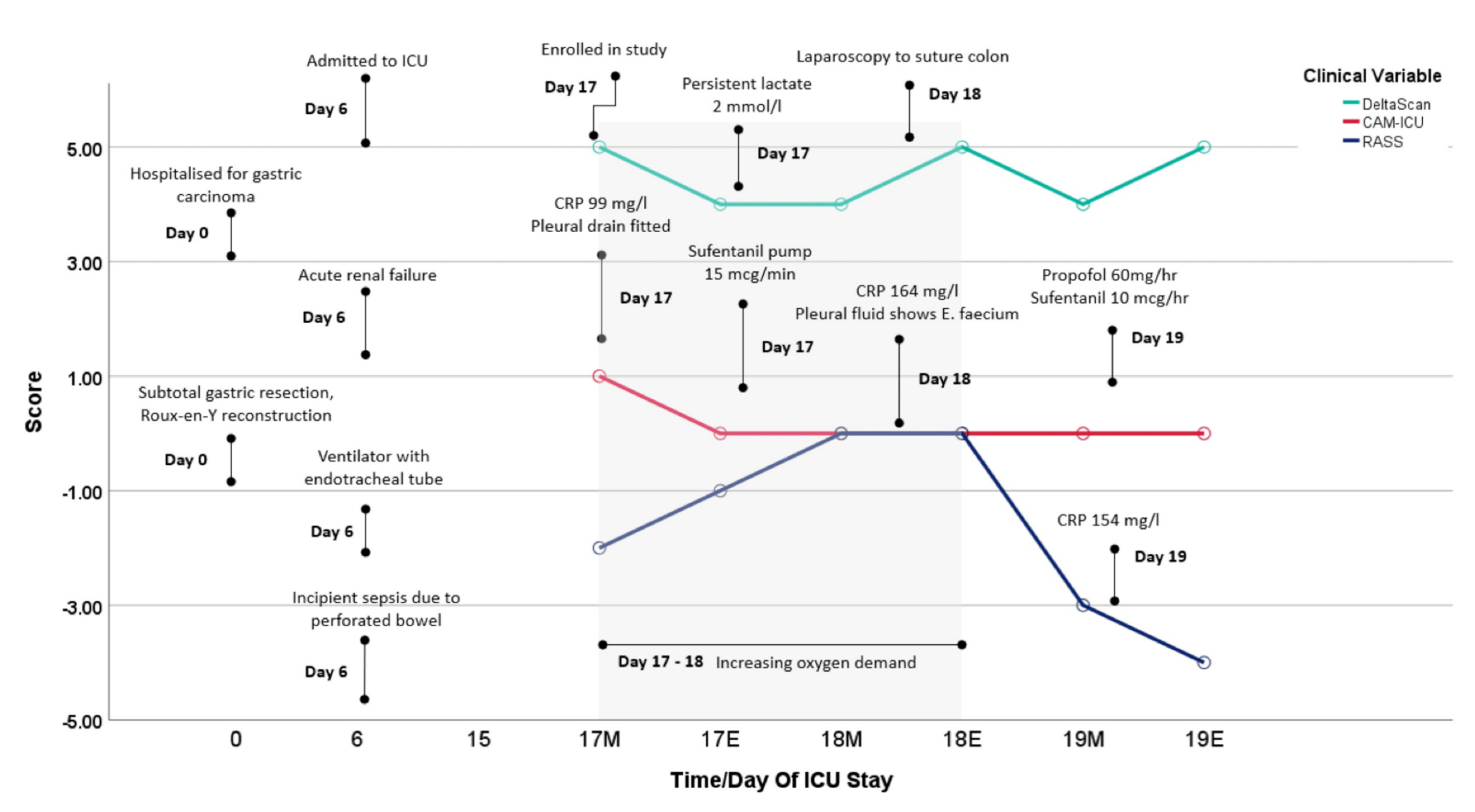
Chronological overview of variable clinical outcomes during the patient's hospitalization Note: The day of the ICU stay is given as well as the time of day: M, morning; E, evening, e.g., 17M = day 17 in the morning. Days 17-19 concern the study period, and measurements were performed twice a day. CRP, C-Reactive Protein

The first measurement point (MP1, 08:00 in the morning) was performed, and sedative medications were discontinued. The RASS score, which needed to be -3 or higher to conduct the CAM-ICU and single-channel EEG, was high enough to conduct the CAM-ICU and the single-channel EEG. The RASS score was -2, and the CAM-ICU was positive for delirium; the DeltaScan indicated a 5, indicating a high probability of delirium/acute encephalopathy (Figure 2). Despite this, as part of usual care, the conclusions of the observer, nurse, and medical team were unanimous in the opinion that the patient was oriented and cognitively adequate and that there were no signs of delirium.

During the first day of measurements, he had an increasing oxygen demand despite interventions from the previous day. The previous day, an X-ray of his chest revealed pleural fluid. Alteplase was rinsed, after which a lot of pleural fluid production started. Pleural fluid was sent to the clinical chemistry lab for further testing. By day one of the study, pleural drain production had returned to normal, and abdominal drain production was minimal. The C-reactive protein (CRP) levels had dropped to 99 mg/L (normal value: <10 mg/L). Analysis of the pleural fluid revealed the presence of *Pseudomonas*, *Enterococcus faecalis*, and *Enterococcus faecium*. The pulmonologist determined that no lobectomy was necessary, but he started the patient on vancomycin. In addition, the patient had a persistent lactate around 2 mmol/L (normal value: 0.5 and 1.5 mmol/L) despite an improving glomerular filtration rate (GFR). During the day, our patient had no fever, had a low norepinephrine requirement (0.039 mcg/kg/minute), and had a good diuresis of 100 cc/hour. Due to pain complaints, a sufentanil pump was running at 15 mcg/minute.

At 3:00 PM, MP2 was performed for the study. The conclusion of the observer, nurse, and medical team was unanimous in the opinion that the patient still exhibited no signs of a delirium despite the single-channel EEG giving a high score (5) for the presence of acute encephalopathy and/or delirium.

On day two, MP3 and MP4 were performed again at 8:00 AM and 3:00 PM. The patient scored noticeably high scores (4 or 5) using the single-channel EEG, while the CAM-ICU was scored as non-delirious by the medical and nursing staff. An increase in oxygen demand was again observed on this day, during which the ventilation settings were changed; the positive end expiratory pressure (PEEP) and the fraction of inspired oxygen (FiO_2_) were increased. Haemodynamically, the patient needed less support, with noradrenaline discontinued and diuresis at 100 cc/hour. At the 12:00 checks, faeces-like substances from abdominal drains were found. A lot of *E. faecium* was cultured from the sent abdominal fluid material. The blood test showed an increased CRP of 99 mg/L and later 164 mg/L (normal value: <10 mg/L) and a decreased GFR of 57 ml/minute and later 39 ml/minute (normal value: >90 ml/minute). After consultation with the abdominal surgeon, the patient underwent a laparoscopy at 4:00 PM, during which a defect of the colon was sutured, and the abdomen was rinsed. One extra drain was also placed in the colon area.

Despite this major operation, the measurements for the study could continue the next day. However, MP5 and 6 were performed at 3:00 PM and 11:00 PM this time. The patient was still on a low dose of sedative medication (propofol 60 mg/hour and sufentanil 10 mcg/hour) and had a low dose of noradrenaline, 0.039 mcg/kg/minute, for low blood pressure. At MP5, a RASS score of -3 was sufficient to be able to perform new measurements. Throughout day three, DC continued to receive high scores (4-5) on the single-channel EEG DeltaScan, while the CAM-ICU was again scored as non-delirious. The ventilation settings and circulatory support remained unchanged between MP5 and MP6. The pleural drain and abdominal drains produced different substances; minimal faecal substance was still detectable from one abdominal drain.

## Discussion

The main objective of this case report was to demonstrate that PMD waves measured by a single-channel EEG could serve as an early detection of potential sepsis. This finding requires validation through high-quality, large-sample studies; however, other studies yielded similar results to ours [13]. The EEG measurement in this case showed consistently high scores, which are calculated by the algorithm in the DeltaScan device. The higher the score, the more PMD waves and thus more slowing in EEG activity.

As this is a case example, we emphasize that more evidence and larger sample sizes are needed to determine whether a single-channel EEG can detect the encephalopathy earlier, resulting from a septic episode, as PMD waves are also present during SAE [12]. The single-channel EEG has been shown to have good detection of acute encephalopathy and of PMD waves, which have been confirmed by other researchers to be present during episodes of sepsis [9,12-14]. Our case illustrates discrepancies between the patient's behavioural data and EEG findings. Notably, the patient exhibited no signs of delirium but progressively deteriorated over time [15-20].

These findings show positive agreement with the available literature, but more research is needed to further evaluate these data so that sepsis treatment may be initiated earlier [15-20]. Future research should focus on increasing the sample size, comparing EEG patterns in SAE and delirium, and refining the diagnostic criteria for SAE [13]. These efforts can contribute to the development of a reliable method for early sepsis detection and improved patient outcomes through timely intervention.

## Conclusions

We reported the clinical course of a complicated surgical patient in the ICU. We found that single-channel EEG and the presence of PMD waves might be helpful in the early recognition of sepsis. Although these findings are not yet substantiated by larger studies, they indicate a potential use of single-channel EEG in potentially detecting sepsis in an early phase.
